# A Framework for Minimally Invasive Remote Robotic‐Assisted Surgery: Bridging Innovation and Patient Safety

**DOI:** 10.1002/wjs.70146

**Published:** 2025-10-22

**Authors:** Yuman Fong, Steven D. Schwaitzberg, Kate Petty, James Porter, Peter G. Schulam, Piet Hinoul, Dennis Fowler, Jaime A. Wong, Louis Kavoussi, Jordana Bernard

**Affiliations:** ^1^ Department of Surgery City of Hope Duarte California USA; ^2^ Department of Surgery Department of Biomedical Informatics University at Buffalo—Jacobs School of Medicine and Biomedical Sciences The State University of New York Buffalo New York USA; ^3^ Sovato Santa Barbara California USA; ^4^ Swedish Medical Center Seattle Washington USA; ^5^ Medtronic Boston Massachusetts USA; ^6^ Johnson & Johnson MedTech Johnson & Johnson New Brunswick New Jersey USA; ^7^ Virtual Incision Lincoln Nebraska USA; ^8^ Intuitive Surgical Inc. Sunnyvale California USA; ^9^ Zucker School of Medicine Hofstra/Northwell Northwell Health New York New York USA

**Keywords:** remote robotic procedure, remote robotic surgery, remote surgery, telementoring, teleprecepting, telepresence, teleproctoring, telestration, telesurgery

## 1 Introduction

The development of scalable remote robotic‐assisted surgery is a potentially transformative milestone in the delivery of surgical care. Remote robotic surgery offers care networks the opportunity to optimize deployment of high‐quality surgical expertise, to minimize patient travel and inconvenience, and to maintain best outcome. It also holds promise as a means of reducing healthcare disparities globally [1].

Using modern advances in robotic and tele‐communication technologies, investigators have established the feasibility of remote robotic‐assisted surgery (RAS) in which the surgeon is not physically in the same location as the patient [2, 3, 4, 5]. These pre‐clinical and clinical studies underscore the need for a framework to guide healthcare stakeholders and medical societies in the development of clinical practice guidelines, with the overarching aim of ensuring the safe and effective integration of remote RAS into routine patient care [5, 6, 7, 8, 9, 10]. Key clinical and operational issues are outlined below for consideration. Relevant terms and definitions are provided in the Appendix A.

## 2 Operating Models

Several distinct operating models are defined by the level of responsibility assigned to the surgeon(s) and other credentialed practitioners involved in the remote RAS procedure [8, 11, 12]. The primary surgeon must be clearly designated prior to engaging in patient care.

These operating models include:

*Telementoring/Teleprecepting without instrument control*—A fully trained and credentialed remote surgeon observes and mentors a patient site credentialed practitioner by providing verbal/visual guidance, including telestration. The telementor has no control of the robotic instruments.

*Telementoring/Teleprecepting with instrument control*—A fully trained and credentialed remote surgeon provides a patient site credentialed practitioner with (1) supervisory and educational verbal–visual guidance, including telestration, and/or (2) by taking control of the tools to demonstrate and/or complete a segment of the RAS procedure.

*Remote teleproctoring*—A qualified remote surgeon observes, evaluates, and reports on the skills and performance of a patient site practitioner.

*Remote robotic‐assisted co‐surgery*—A fully trained and credentialed remote surgeon collaborates with a fully trained patient site credentialed practitioner to complete a remote‐enabled robotic procedure. Each surgeon/practitioner completes the part of the procedure for which they are credentialed.

*Full remote robotic‐assisted surgery*—A fully trained and credentialed remote surgeon performs remote RAS as the primary surgeon with a credentialed practitioner as the bedside assistant. In this configuration, the remote surgeon retains primary responsibility for the patient's surgical care.

## 3 Care Team Roles and Qualifications

The remote RAS care team must be similar to a typical RAS team, with at least one surgeon remote from the patient site. The team at the patient site should include at least one site credentialed practitioner, a circulating nurse, a scrub nurse/technician, and an anesthesiologist/credentialed nurse anesthetist (CRNA). Depending on the operating model, the patient site practitioner could be the primary surgeon (and mentee), co‐surgeon, or bedside assistant. Appropriate credentialing is required for each role. Each team member must have foundational expertise in RAS procedures and specialized training in remote surgery, with an emphasis on team coordination and the management of crises unique to remote RAS [7, 8, 12].

## 4 Clinical Pathway

Appropriate patient selection is critical to ensuring both patient safety and the success of a remote RAS program. Consistent with current practice, patient selection for remote RAS should be guided by comprehensive risk assessment that accounts for both patient‐specific factors and the capabilities of available resources. The surgeon with primary operative responsibility for the patient must conduct a pre‐operative consultation, either in person or virtually. This discussion should include the options of nonsurgical care, open surgery, laparoscopic surgery, and local RAS (See Section 6).

Using a telepresence system, a pre‐operative technical time out should be completed prior to the induction of anesthesia to document connectivity and functionality of robot system, as well as the patient and procedure. Subsequently, a traditional time out including the pre‐operative safety checklist must then be completed prior to incision. All listed surgeons, including the remote surgeon, should participate and attest to the time out either in person or remotely.

Surgical access and port placement should follow standard protocols.

At the conclusion of the remote surgeon's involvement, the entire care team must complete a sign‐out checklist.

Post‐operatively, patients must receive clear discharge instructions, including contact information for the responsible practitioner. Instructions should outline contingency plans for urgent or emergency scenarios and provide guidance for follow‐up care. If the patient requires hospital admission or overnight recovery, the admitting service must be designated and have access to contact information for the remote or primary surgeon.

## 5 Emergency Management

The entire remote RAS care team must be prepared to manage serious adverse events or crises and should begin that preparation during the time out by discussing the risk levels for bleeding and conversion, as well as the potential need and availability for additional equipment, blood products, back‐up surgical expertise, or the need for transfer. An emergency communication alternative should be identified, tested, and clearly available to the team [7, 8, 10, 12].

The roles and responsibilities of each member of the remote RAS care team should be clearly defined for each potential crisis. Responses to specific technology failures should mirror those already defined and implemented for crises during local RAS. In the event of a power failure, network disruption, or RAS device malfunction, the patient site care team, led by the patient site credentialed practitioner, should determine whether to prioritize emergency conversion or troubleshoot the technology. This decision should be based on rapid assessment of: (1) the urgency of continuing the intervention, (2) the estimated time to restore the function of the technology, and (3) the availability of personnel and equipment to complete the RAS procedure. Ultimate responsibility for managing a serious adverse event in the operating room during a remote RAS procedure lies with the patient site care team.

## 6 Medico‐Legal and Ethical Considerations

### 6.1 Informed Consent and Medical Liability

During the informed consent process, patients offered the option of remote RAS must receive the same comprehensive information as those undergoing local RAS procedures.

Informed consent for remote RAS must also include disclosure of the relevant operating model and the following details:

1. Practitioner roles and locations
  - The roles and locations of each practitioner and the possibility that the remote surgeon may not be physically present in the operating room.
  - Initial incision(s) may be performed by a credentialed practitioner, other than the primary surgeon.
  - The remote surgeon will have access to the patient's medical records.
2. Risks specific to remote RAS
  - Potential for technology failure
  - Possibility that a different surgeon, who may be less familiar with the case, may be needed to complete the procedure.
  - Cybersecurity risks, including breaches of personal health information.
3. Emergency protocols
  - Patients must be informed of the emergency response plan, including who will assume responsibility if the primary surgeon is not available.
  - Patients requiring transfer to another facility must be informed of the receiving location and physician.

A written agreement defining medical responsibility must be executed by the participating institutions and providers, including both local and remote surgeons.

Medical liability and responsibility for adverse outcomes after or during remote RAS should be actively discussed and negotiated among stakeholders. For remote surgeons, liability may be tied to their role in directing the procedure, regardless of whether they are physically controlling the instruments. Additional factors influencing liability should be explored collaboratively by the relevant stakeholders. Malpractice insurance coverage for remote surgery should reflect these dynamics, ensuring that remote surgeons are adequately protected based on their clinical responsibilities, despite being physically absent in the operating room [9, 13].

### 6.2 Standard of Care

Remote RAS must meet or exceed the local standard of care, defined as the level and type of care that a reasonably competent and skilled healthcare professional, with a similar background and working in the same medical community, would provide under the same or similar circumstances.

### 6.3 Credentialing and Delineation of Privileges

The remote surgeon, the local surgeon or credentialed practitioner, as well as the supporting staff, must be formally trained in the remote use of the RAS device that has been approved for remote procedures (See Section 3 Roles and Responsibilities).

### 6.4 Ethical Considerations

Remote RAS offers a transformative approach to surgical care that aligns with the principles of the quintuple aim [14]:

1. Enhancing patient experience
2. Improving population health
3. Reducing the cost of care
4. Supporting remote RAS care team experience
5. Advancing health equity

From a deontological ethical perspective, particularly through the lens of Beauchamp and Childress's Four Principles of Biomedical Ethics—autonomy, beneficence, nonmaleficence, and justice—ethical considerations arise when evaluating remote RAS [2, 7, 9, 12, 13, 15]. Remote RAS can be ethically justified if it upholds key moral obligations [15].

These ethical obligations must also be interpreted in the context of evolving technology, practice standards, and disparities in access to care. Ethical justification requires transparency and mechanisms for accountability including clear delineation of responsibility among providers and institutions [7, 8, 12].

## 7 Cross‐Border Considerations

Remote RAS introduces multi‐jurisdictional legal and regulatory challenges that span health systems, regions, and countries [2, 3, 7, 8, 9, 12, 13]. Delivering care across borders requires remote surgeons to comply with a wide range of laws, regulations, and institutional policies, including those governing licensure, scope of practice, and credentialing and privileging requirements in the patient's jurisdiction. The remote RAS system must also be cleared by the regulatory authority overseeing the patient site and, in some cases, by the authority in the remote surgeon's country of practice. Additional considerations include adherence to international telemedicine frameworks related to privacy, data protection, and cybersecurity as well as customs and import requirements for medical equipment.

Reimbursement policies vary across borders and jurisdictions. Sustainable models will require close collaboration with policymakers and payers to develop reimbursement pathways that recognize the clinical value, operational complexity, and quality standards of remote RAS [16].

### 7.1 Role of Surgical Societies

Surgical societies should play an instrumental role in evolving clinical guidelines for remote RAS programs [7, 12]. Surgical societies representing each surgical discipline should align on core principles for remote RAS and modify to meet discipline‐specific and procedure‐specific requirements. Surgical societies should also play a role in developing education and advanced experiential training to prepare surgeons for safe and effective practice in remote RAS.

## 8 Technical Considerations

The technical infrastructure that enables remote RAS is multi‐faceted and includes a surgical‐grade network, telepresence system to restore situational awareness for the care team members, and software that supports safe workflow [2, 7, 8, 10, 12]. The network must provide high reliability, ultra‐low latency, and robust cybersecurity. The telepresence system must facilitate routine verbal and visual communication between the members of the care teams at both sites while workflow management must ensure secure connection of remote controllers to devices.

Both the remote surgeon site and the patient site must have the appropriate network and telepresence infrastructure, and the patient site must meet the same space requirements as local RAS procedures. Each site must also have access to trained technical support for system performance testing, maintenance and management before, during and between procedures.

To support the safe expansion of remote surgical programs, an industry‐led coalition developed expert consensus‐based technical guidelines for remote surgery and procedures [10]. These guidelines inform the design and implementation of the infrastructure for establishing a safe, effective, and scalable remote robotic programs. What is needed now is a gathering of clinicians to produce a similar consensus clinical guideline for implementation of remote robotic surgery.

## Author Contributions

**Yuman Fong:** conceptualization, writing – original draft, writing – review and editing. **Steven D. Schwaitzberg:** conceptualization, writing – original draft, writing – review and editing. **Kate Petty:** conceptualization, writing – original draft, writing – review and editing. **James Porter:** conceptualization, writing – original draft, writing – review and editing. **Peter G. Schulam:** conceptualization, writing – original draft, writing – review and editing. **Piet Hinoul:** conceptualization, writing – original draft, writing – review and editing. **Dennis Fowler:** conceptualization, writing – original draft, writing – review and editing. **Jaime A. Wong:** conceptualization, writing – original draft, writing – review and editing. **Louis Kavoussi:** conceptualization, writing – original draft, writing – review and editing. **Jordana Bernard:** conceptualization, writing – original draft, writing – review and editing, project administration.

## Conflicts of Interest

Y. Fong declares scientific consultant for Medtronic, Johnson & Johnson, and Imugene, receives royalties from Merck and Imugene, owns patents for CF33‐OVs and CF33‐CD19t licensed to Imugene LTD. S.D. Schwaitzberg declares Stryker Endoscopy consulting fees, University of South Alabama, grand rounds honorarium, MILMIC, medical malpractice testimony, Academic Health Insurance board member, Sages Ingenuity VP, Sovato Advisory Board and stock options, HIA Technologies stock. K. Petty declares Sovato stock options. J. Porter declares Medtronic stock options. P.G. Schulam declares stock. P. Hinoul declares Virtual Incision stock or stock options. D. Fowler declares Sovato stock options. J.A. Wong declares Intuitive Surgical stock and stock options. L. Kavoussi declares Sovato Advisory Board. J. Bernard declares Sovato stock options.

## Appendix A Terms and Definitions

The terms and definitions listed below are used to support clarity and consistency. We acknowledge that practitioners and organizations may use different terminology or interpret these concepts differently, and that some variation and differences of opinion may exist within the field.

*Bedside proceduralist:* A practitioner credentialed to assist at the patient's bedside during RAS performed by the primary surgeon at the RAS device console. Examples of practitioners who could be qualified to be a bedside proceduralist include a surgeon, physician, PA, NP, or RNFA with additional training to specifically assist during RAS procedures.

*Credentialed practitioner:* A provider credentialed in accordance with the applicable healthcare system's policies and procedures. Examples include a surgeon, physician, PA, or NP.

*Emergency response practitioner:* A credentialed practitioner who is available and qualified to address a surgical emergency at the patient site. In these circumstances:

*Full remote robotic‐assisted surgery:* A remote surgeon performs remote RAS as the primary surgeon who has full responsibility for the patient. In this configuration, the remote surgeon retains sole responsibility for all aspects of the patient's surgical procedure.

*Hard stop:* A forced stoppage of workflow to determine the best next steps.

*Latency:* The time measurement between when a source transmits data and the destination receives the data.

*Patient site:* The operating room or facility where the patient undergoes the remote RAS procedure.

*Patient site care team*: The care team at the patient site supporting the remote procedure (e.g., nurses, OR technicians, anesthesiologists, clinical engineers, and other physicians).

*Patient site credentialed practitioner:* A provider credentialed in accordance with the applicable healthcare system's policies and procedures. Examples include a surgeon, physician, PA, or NP.

*Primary surgeon:* The credentialed practitioner who holds ultimate responsibility for the patient throughout the entire surgical process, including pre‐operative, intra‐operative, and post‐operative care.

*Remote‐enabled robotic system*: This refers to the entire system used for remote procedures, including the robotic system at the patient site, the physician's console at the remote surgeon site, and any hardware or software needed to enable a safe and effective procedure over a surgical‐grade network connection. Also known as “Remote RAS Device.”

*Remote‐enabled robot*: A RAS device that has been modified to enable its console to communicate with the bedside tower across a network.

*Remote robotic‐assisted co‐surgery*: A fully trained and credentialed remote surgeon collaborates with a fully trained patient site credentialed practitioner to complete a remote RAS procedure. Each surgeon/practitioner completes the part of the procedure for which they are credentialed.

*Remote robotic‐assisted surgery or procedure:* A clinical procedure in which a physician uses a remote‐enabled RAS device and surgical‐grade network to perform a procedure on a patient in a different location. Also known as “remote RAS,” “remote surgery,” “remote procedure,” and/or “telesurgery.”

*Remote surgeon*: A surgeon who is in a different location from the patient.

*Remote surgeon site*: The room or facility in which the remote surgeon performs remote RAS.

*Session:* This refers to a single instance of a remote RAS procedure encompassing the time from when the connection is established to when it is terminated.

*Surgical‐grade network*: A managed network with high reliability, robust security, and guaranteed quality of service, which supports the requirements of remote procedures as outlined in the expert consensus‐based technical guidelines for remote surgery and procedures.

*Telementoring/Teleprecepting without instrument control:* A fully trained and credentialed, as required, remote surgeon observes and mentors a patient site credentialed practitioner by providing verbal/visual guidance, including telestration. The telementor has no control of the robotic instruments.

*Telementoring/Teleprecepting with instrument control:* A fully trained and credentialed remote surgeon provides a patient site credentialed practitioner with (1) supervisory and educational verbal–visual interactions, including telestration, and/or (2) guidance by taking control of the tools to demonstrate and/or complete a segment of the RAS procedure.

*Telepresence*: The use of audio–video technology to enable an individual to be virtually in another site.

*Teleproctoring*: A qualified remote surgeon observes, evaluates and reports on the skills and performance of a patient site practitioner.

*Time out*: The surgical team's short pause, just before induction of anesthesia and/or just before incision, to confirm that all required equipment is available and functional, and that the team is about to perform the correct procedure on the correct body part of the correct patient in the right facilities.

*Remote Surgery/Procedure use case:* The types or categories of procedures that practitioners perform as part of a remote surgery or remote procedure program, such as remote minimally invasive surgery, remote endovascular procedures, remote interventional procedures, remote endoscopy, or other diagnostic procedures that may be performed remotely. Telementoring with instrument control may be a component of any of these use cases.
